# School eHealth Education Program Pakistan (eSHEPP): An Exploratory Qualitative Study of Stakeholder Perspectives on Design, Barriers, and Facilitators

**DOI:** 10.21203/rs.3.rs-7735852/v1

**Published:** 2025-09-30

**Authors:** Muhammad Shahid Khan, Aysha Almas, Zainab Samad, Kanecia Obie Zimmerman, Tazeen Saeed Ali

**Affiliations:** Aga Khan University; Aga Khan University; Aga Khan University; Duke University; Aga Khan University

**Keywords:** adolescent health, digital health, health education, schools, Pakistan, noncommunicable diseases, eHealth, qualitative research

## Abstract

**Background:**

Noncommunicable diseases (NCDs) are a growing health challenge in low- and middle-income countries (LMICs), including Pakistan. Adolescence is a critical period for shaping lifelong behaviors, yet school-based health education remains limited and inconsistently implemented. Digital health interventions offer scalable opportunities, but their feasibility, sustainability, and cultural acceptability in LMIC school settings remain underexplored.

**Objective:**

This study examined barriers and facilitators to delivering the School eHealth Education Program Pakistan (eSHEPP) and explored stakeholder perceptions of its design, delivery, and content for adolescent NCD prevention.

**Methods:**

An exploratory qualitative design was applied in public secondary and higher secondary schools using purposive sampling. Data were collected through four focus group discussions with students and teachers (N=36) and 11 key informant interviews with parents and administrators. Interview guides were informed by the Technology Acceptance Model and the Task–Technology Fit framework. Transcripts were thematically analyzed in NVivo v14 using a hybrid deductive–inductive approach. Credibility was supported through intercoder reliability (κ=0.71) and stakeholder validation.

**Results:**

Major barriers included infrastructure gaps such as unreliable internet, electricity shortages, and lack of multimedia resources. However, students’ digital familiarity and widespread mobile access were strong facilitators. Parents, teachers, and administrators endorsed eSHEPP, noting students’ enthusiasm and the spillover of health knowledge to families. Stakeholders recommended a bilingual (Urdu/English), offline-accessible app with intuitive navigation, privacy safeguards, and interactive tools such as quizzes and rewards. Short Urdu videos with English subtitles, relatable scenarios, and student involvement were considered most engaging. Cultural sensitivities around mental health, gender norms, and substance use require careful framing. Sustainability was viewed as dependent on curriculum integration, teacher training, and institutional support.

**Conclusions:**

eSHEPP shows strong potential as a culturally sensitive, scalable, and pedagogically sound model for adolescent health promotion in LMIC schools. Addressing infrastructural gaps, ensuring policy integration, and promoting digital equity will be critical for long-term impact.

## Introduction

Noncommunicable diseases (NCDs) and their risk factors are major global public health concerns, particularly in low- and middle-income countries (LMICs), where they account for a disproportionate burden of morbidity and mortality (1, 2). Adolescents are especially vulnerable, as many risk behaviors that contribute to NCDs, including physical inactivity, substance use, poor diet, and overweight or obesity, are established during this stage of life (3–5). These behaviors have long-term consequences, with research showing that a large share of premature adult deaths are linked to habits formed in adolescence (4, 5). Recognizing this, the World Health Organization (WHO) has emphasized adolescent NCD prevention as a public health priority and called for innovative approaches to reach young people effectively (6, 7).

Schools are uniquely positioned to promote adolescent health. As a primary setting where young people spend much of their time, schools offer a platform for structured health education, skill-building, and behavior change (8, 9). Evidence from LMICs shows that school-based interventions can successfully improve students’ nutrition, physical activity, and other health-related behaviors (10, 11). However, in Pakistan, school-based health education remains underdeveloped. Health topics are rarely part of the formal curriculum, and when present, they are often limited to didactic lectures that provide information but do not engage students in meaningful discussion or practical skills (12, 13). Several systemic barriers further limit the effectiveness of health education in Pakistani public schools. These include inadequate infrastructure, limited teaching resources, and a shortage of instructors trained in adolescent health (14). Cultural sensitivities also restrict discussion of topics such as mental health, reproductive health, and gender, leaving many students without access to accurate information or supportive dialogue (13, 15). As a result, adolescents often lack the knowledge and confidence needed to make informed health choices, and schools remain an underutilized channel for NCD prevention.

Digital interventions offer an innovative, accessible, and cost-effective way to address these gaps. With the widespread rise in mobile phone usage across Pakistan (16), digital platforms provide promising opportunities to deliver engaging and contextually relevant health content to adolescents (17, 18). Children and adolescents are already active users of mobile applications, social media, and online platforms, which have been leveraged globally for peer-to-peer education, health promotion, and mental health support (19–22). Emerging evidence shows that eHealth interventions can improve health literacy, encourage positive behavior change, and extend the reach of prevention programs, particularly in resource-constrained settings like Pakistan (17, 20, 21, 23–27). However, the success of such programs depends on how well they reflect the daily lives, technological access, and cultural contexts of their intended users. Interventions developed without this grounding risk a “design–reality gap,” leading to low uptake and disengagement (28–30), while those shaped by stakeholder input achieve stronger engagement and more sustainable impact (28, 31, 32).

Formative qualitative research is therefore essential to guide the design and delivery of school-based eHealth programs in Pakistan. By capturing the perspectives of students, teachers, parents, and school administrators, researchers can ensure that interventions are not only contextually appropriate but also feasible to implement at scale within the constraints of the public education system. To respond to this need, we developed the School eHealth Education Program Pakistan (eSHEPP) to promote adolescent health literacy and reduce NCD risk behaviors. This exploratory qualitative study examined barriers and facilitators to eSHEPP delivery in public secondary and higher secondary schools and assessed stakeholder perceptions of its design and content. To our knowledge, this is the first study in Pakistan to qualitatively explore stakeholder perspectives on a school-based eHealth program for adolescents. Findings will guide refinements to eSHEPP and support the integration of digital health education into Pakistan’s education and public health systems, with lessons for other LMICs.

## Methods

An exploratory qualitative study was conducted in Karachi, Pakistan to identify barriers and facilitators affecting the delivery of eSHEPP in secondary and higher secondary schools, and to capture stakeholders’ perspectives on the program’s design and content.

### Conceptual Framework for Developing Key Informant Interviews (KIIs) and Focused Group Discussion (FGD) Guides

**Figure 1:**
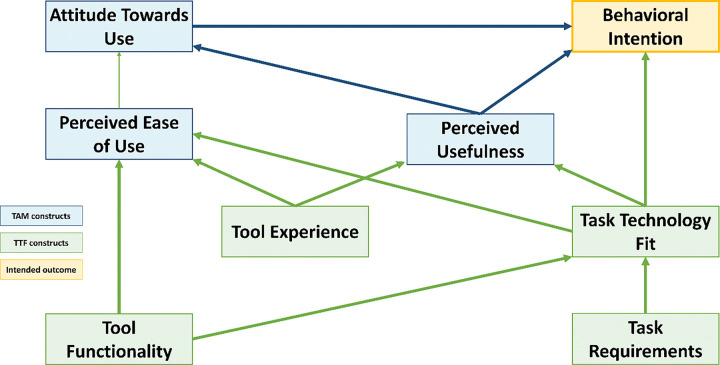
Integrated model of TAM and TTF

This exploratory qualitative study was guided by an adapted conceptual framework that integrates the Technology Acceptance Model (TAM) and the Task–Technology Fit (TTF) (33). The framework was used to explore users’ perceptions of the eSHEPP program’s design, with a particular focus on perceived usefulness, perceived ease of use, and attitudes toward use. These components provided a structured approach to understand key user insights relevant to the effective development of the eSHEPP intervention. Figure 1 shows the conceptual framework guiding the development of the FGD and KII guides, which are provided in S5 File.

### Sampling Technique and Participants

A purposive sampling strategy was employed to ensure diverse representation across stakeholder roles, experience levels, and availability. Participants were recruited through school principals, who facilitated initial contact with students, teachers, and parents, while school administrators were directly approached for interviews.

Inclusion criteria for participants were as follows: students in grades 9–12, teachers with at least six months of teaching experience in relevant schools, parents or guardians of enrolled students, and school administrators, including principals, vice principals, District Education Officers (DEOs), and officials from the Provincial Department of Education.

Four FGDs were conducted: two with students (one male, one female) with 10 participants in each group, and two with teachers (one male, one female) with 8 participants in each group. In addition, 4 KIIs were conducted with parents and 7 KIIs with school administrators to gather in-depth insights from those in caregiving and leadership roles. In total, 47 participants were included: 20 students, 16 teachers, 4 parents, and 7 school administrators. Participants for FGDs and KIIs were purposively selected to ensure diversity in gender, experience, and school type. School principals facilitated the initial identification of potential participants, and the research team confirmed eligibility based on the inclusion criteria. One parent and two administrators declined participation due to time constraints. No individuals other than participants and researchers were present during data collection. Detailed demographic characteristics of the study sample are provided in S3 File (Tables S4–S7).

### Data Collection

Semi-structured interview guides were developed using the integrated TAM–TTF framework and organized into four sections: general perceptions of digital health applications (TAM: perceived usefulness and ease of use), barriers to implementation (TTF: students’ learning needs, engagement, and contextual challenges), facilitators to implementation (factors supporting delivery), and program content and design (perspectives on videos and application features). The guides were initially prepared in English, translated into Urdu, and pilot tested (two FGD and two KIIs) to ensure clarity and cultural relevance. At the time of data collection, participants had no direct exposure to eSHEPP; their perspectives were gathered to guide program design and adaptation before school-based delivery.

FGDs, lasting 45–60 minutes, and KIIs, lasting 20–30 minutes, were conducted sequentially in school activity rooms or stakeholder offices. Each session began with informal conversation to build rapport and explain the study purpose, and discussions were audio-recorded and supplemented with field notes. Written informed consent was obtained from all participants, with both parental consent and student assent secured for those under 18.

### Research Team and Reflexivity

All FGDs and KIIs were conducted by the lead researcher (MSK, male, PhD Candidate (Population and Public Health, Aga Khan University), formally trained in qualitative methods with prior community-based research experience. No prior relationships existed with participants; contact was facilitated through school administrators. For cultural sensitivity, the male researcher conducted FGDs and KIIs with male participants, while a trained female research assistant facilitated those with female participants. Reflexivity was maintained through ongoing debriefs with the research team to critically examine assumptions and potential biases.

### Data Management and Analysis

Data collection continued until thematic saturation was reached. Saturation was defined as the point at which no new codes or themes emerged across two consecutive FGDs or KIIs. Audio recordings were transcribed verbatim and cross-verified by the lead researcher within two weeks. Data were analyzed using a hybrid coding approach: deductive coding based on TAM–TTF constructs and inductive coding for emergent themes.

The codebook underwent six iterative development cycles over three months, with team discussions refining parent–child codes and ensuring alignment with the integrated TAM–TTF framework (see S1 File: Tables S1–S3). Two independent coders analyzed the transcripts. Intercoder reliability was assessed on a subset of transcripts (25%), yielding substantial agreement (Cohen’s κ = 0.71, p < .001). A detailed coding tree is available in S3 File (Figure S2). Thematic validation was achieved through team discussions, and representative quotes were selected to illustrate key findings. To enhance credibility, a stakeholder validation meeting was conducted where preliminary findings were presented and discussed, allowing participants to provide feedback on interpretation. Data were managed and analyzed using NVivo v14. The study adhered to the Consolidated Criteria for Reporting Qualitative Research (COREQ) checklist throughout (see S4 File).

### Ethical Approval

The study received ethical approval from The Aga Khan University Ethical Review Committee (Ref No. 2023–9277-27367). Any significant amendments were subject to approval by the project’s steering committee and re-submission to the ethics committee.

## Results

**Figure 2:**
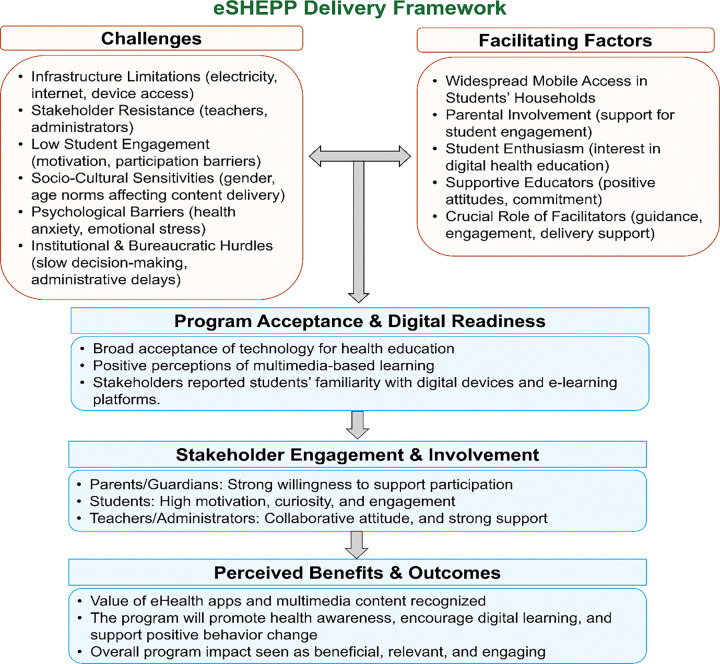
eSHEPP delivery framework for school students aged 13–18 years

Stakeholders identified key challenges to eSHEPP, including limited infrastructure, resistance to change, sustaining student engagement, and cultural sensitivities. Reported enablers included widespread mobile access, parental encouragement, student motivation, and supportive teachers and administrators. Active involvement across stakeholder groups was considered essential, with participants demonstrating readiness for digital learning through openness to multimedia tools and confidence with technology. These findings informed the proposed eSHEPP delivery framework (Figure 2).

### Anticipated Challenges

Stakeholders highlighted multiple barriers to program delivery. **Infrastructural limitations** were most frequently cited, including lack of multimedia equipment, unreliable electricity, and poor internet access. As Administrator 2 explained, *“Most government schools do not have multimedia systems,”* while Administrator 1 added, *“A dedicated activity or multimedia room is essential… not all schools have these resources.”* Electricity and connectivity were recurring concerns: *“There is a significant issue with electricity”* (Administrator 7), and teachers estimated that *“Only five to ten percent of schools might have [internet access]”* (FGD-T1).

Challenges with **stakeholder buy-in** were also noted. Resistance from senior teachers was common; Administrator 1 remarked, *“Some older teachers are rigid and disinterested, viewing sessions as a waste of time.”* Similarly, principals’ support varied: *“Some principals cooperate, others do not. Convincing them is part of your role”* (Administrator 2).

Maintaining **student engagement and motivation** was considered another obstacle. Administrator 4 highlighted absenteeism: *“Absenteeism is a significant challenge… extraordinary steps are needed to ensure attendance.”* Administrator 5 added, *“Children are generally not inclined to participate in such activities nowadays; you will need to motivate them effectively.”*

**Socio-cultural sensitivities** were also emphasized, particularly regarding gender norms and religious boundaries. Parent 3 explained, *“In our Pakistani culture, there are certain boundaries… doing so can create awkwardness, especially for females.”* Administrator 3 noted, *“Some matriculation students may prefer not to receive information in a particular way due to cultural practices, such as wearing a niqab.”*

Stakeholders further cautioned against **psychological impacts** of health education, warning that poorly framed content could induce fear or anxiety. Administrator 3 stated, *“Raising awareness may generate anxiety, and you need to know how to deal with it.”* Teachers similarly noted, *“Medical information can cause unnecessary worry… it is important that students act appropriately without becoming anxious”* (FGD-T2).

**Institutional and external disruptions** were acknowledged. Parent 3 highlighted bureaucratic hurdles: *“Approval from higher authorities is required, but once secured, they can influence students.”* Teachers also warned of interruptions from unplanned events: *“A sudden government program could halt your entire project”* (FGD-T2).

### Facilitating Factors

Stakeholders highlighted several enablers for the success of eSHEPP. **Access to devices and connectivity** was widely reported, even among low-income families. Administrator 6 remarked, *“Smartphones have reached even the poor… I have seen laborers using them.”* Where broadband was lacking, families relied on affordable mobile data: *“People use mobile data, as affordable packages are available”* (Administrator 6). Teachers often bridged digital gaps by contributing their own equipment: *“We brought our own laptops in previous initiatives… teacher interest is crucial”* (Administrator 7).

**Parental awareness and engagement** were considered vital. Administrator 1 described awareness as “medium,” noting that parents frequently raised concerns about unhealthy food and its links to addiction. Parent 2 emphasized active involvement: *“We even discuss [anti-tobacco ads] with our children,”* and further argued, *“It should have been done at the government level, it will be very beneficial.”*

**Student interest and participation** were also strong enablers. Administrator 1 observed, *“Students… use technology more.”* Students themselves stressed the value of multimedia content: *“These videos will help us learn things not covered in our curriculum”* (FGD-S2). Teachers noted the importance of peer learning: *“If some children don’t understand, others help them… they often became facilitators themselves”* (FGD-T1).

Support from **teachers and administrators** was seen as essential, with the program described as complementary to existing curricula. Teachers explained, *“This complements our science and life skills classes and saves lesson prep time”* (FGD-T1). Administrators recalled prior positive experiences: *“We used to show health content with projectors… it had a strong impact”* (Administrator 2). Teachers also highlighted feasibility: *“Taking 20 to 30 minutes weekly is manageable”* (FGD-T2).

The **facilitator’s role** was emphasized, particularly in resource-limited schools. Administrator 2 noted, *“Most government schools lack multimedia, but your representative could bring it and prepare them.”* Students were described as especially receptive to external facilitators: *“Students pay more attention when someone from outside visits… change matters”* (Administrator 3). A combined teacher–facilitator model was preferred: *“Teachers should stay involved, so students respond more positively”* (Administrator 5).

### Stakeholder Engagement and Involvement

**Parental involvement** varies by context. Urban families were generally more engaged, while rural and low-income households faced barriers due to livelihood pressures. As Administrator 6 explained, *“Parents are too focused on earning a livelihood to pay attention to such matters.”* Shared devices often encouraged indirect participation: *“When students watch videos, they do so with parents… who automatically get involved”* (FGD-T1).

**Student expectations** centered on short, interactive, and emotionally relatable content. One student remarked, *“If there’s a sad scene, there should be sad music, like in movies”* (FGD-S1). Students also valued creating content, with Administrator 3 noting, *“They get extremely excited seeing their own videos… parents also express satisfaction.”*

**Teacher and administrator engagement** was seen as critical for sustaining eSHEPP. Parent 4 described the cascade of influence: *“If the principal understands the message, they guide teachers, who guide students, and students influence parents… a motivated principal can motivate both teachers and parents.”* Teachers emphasized their role in continuity: *“Teachers can register new students and explain the program each year”* (FGD-T1). School Management Committees (SMCs) were also viewed as effective platforms. Administrator 1 explained, *“If the HM, teachers, and parents are involved, 50% of success is already achieved.”* A triangular model linking teachers, students, and parents was emphasized: *“When teachers are informed, students learn; when students learn, it reaches the parents”* (FGD-T1).

Stakeholders highlighted the importance of **teacher training for sustainability**. Administrator 1 suggested, *“It’s better to train teachers as master trainers and empower them to continue… If school health is part of teacher training, students will more readily accept it.”* Teachers echoed this: *“With proper training and a user-friendly guide, we can run the program independently after it ends”* (FGD-T2).

### Program Acceptance and Readiness for Digital Health Education

Stakeholders expressed strong support for digital tools in health education, citing their accessibility, flexibility, and alignment with students’ media habits. Administrator 3 emphasized, *“Some things can’t be conveyed verbally; multimedia plays a key role.”* Parents echoed this enthusiasm, highlighting feasibility and inclusivity: *“Almost every second household has a phone… an app is definitely needed”* (Parent 2). Parent 3 added, *“Showing videos in schools is a great idea; real-life examples are more effective than textbooks.”*

Multimedia-based content, particularly short videos, dramatizations, and animations, was viewed as engaging and more effective than textbook learning. Administrator 7 noted, *“Children are fed up with textbook knowledge. They want to see everything practically.”* Parent 2 explained, *“When you show them a video… it becomes much clearer. If you just give them the facts to memorize, it is difficult for them.”*

Students across urban and rural schools were described as digitally literate and confident in using smartphones, apps, and online platforms. Teachers confirmed, *“They use gadgets a lot; using apps won’t be difficult.”* (FGD-T1), while also noting prior use for formal learning: *“Their classes were on Google Classroom”* (FGD-T2).Another teacher observed, *“You can assume 100% know how to use digital apps”* (FGD-T2).

Although students and teachers frequently engaged with digital platforms for entertainment or informal learning, structured health education apps were largely unfamiliar. As students shared, *“I used a health and care app… it showed how to maintain one’s body and environment”* (FGD-S1). Others remarked on the lack of local tools: *“I don’t think such an app exists in Pakistan… maybe in America or England”* (FGD-S2).

### Perceived Benefits and Motivation

Participants praised the eHealth app as a flexible, private learning tool with advantages over one-time sessions. Students valued its repeatability: *“The app would be better; we can access information anytime”* (FGD-S2). Parents emphasized institutional credibility: *“We can’t verify other platforms, but if this is from an institution, it’s excellent”* (Parent 4). The app also supported home-based learning, with Administrator 7 noting, *“The child will educate parents at home… many concerns will be addressed there.”*

Health videos were seen as powerful drivers of behavior change, with visual methods preferred over verbal instruction. As teachers noted, *“If they only hear, they may forget; if they see, they remember; if they do it, it becomes expertise”* (FGD-T2). Videos were also considered easy to integrate into classes, as Administrator 1 suggested: *“If we align them with the science period, it will positively influence behavior and attitudes.”*

The program was viewed as timely and culturally acceptable, addressing urgent health needs while reinforcing academics. Parent 4 affirmed, *“There will be no cultural or social barriers… students, teachers, and principals will unite to make it successful.”* A strong ripple effect on families was anticipated, with students seen as effective conduits to households. Administrator 6 shared, *“If children are told, ‘Tell your parents…’ education can reach families.”* Participants also stressed the need for scaling and institutionalization. Administrator 2 concluded, *“Your program should succeed; the government should adopt it through the education department.”*

### Stakeholder Recommendations for eHealth Application and Health-Promoting Videos

**Figure 3:**
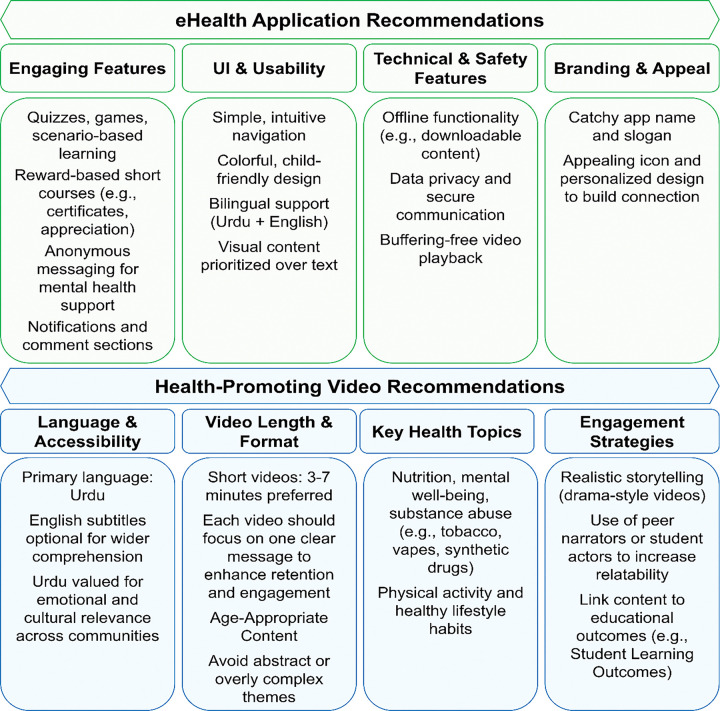
Stakeholder Recommendations for eHealth App and Health-Promoting Videos

Stakeholders recommended a simple, engaging app (English with optional Urdu) with intuitive navigation, interactive features, offline access, privacy safeguards, and appealing branding. They emphasized short Urdu videos with English subtitles on key health topics, delivered through relatable storytelling and student actors to enhance engagement. These recommendations are summarized in Figure 3.

### Recommendations for the eHealth Application

Stakeholders emphasized the value of interactive and reward-based features to sustain engagement. Students proposed, *“There should be multiple-choice questions based on small examples”* (FGD-S1), while Administrator 4 suggested, *“Offer certificates or appreciation letters for participation.”* Gamification was highlighted as a strong motivator: *“Completing one level unlocks the next; we can collect stars”* (FGD-S2). To support mental health, anonymous messaging was proposed; as Administrator 1 noted, *“If problems exist at home, students may not talk to family… it’s easier to message someone anonymously.”* Parents also recommended notifications and comment sections to enhance interaction: *“The comment section is very important,”* and added, *“A notification option would be great”* (Parent 1). Branding was seen as critical, with Administrator 3 advising, *“The app’s name should feel personal… the slogan should be catchy, and the icon appealing.”*

Ease of use was identified as essential, with calls for a simple, child-friendly interface, intuitive navigation, and bilingual support. Administrator 4 emphasized, *“The application should be user-friendly… Keep the language simple.”* Visual elements were preferred over text, as teachers noted, *“Use pictures; they capture children’s attention better”* (FGD-T1).

Participants identified technical and safety concerns. Students suggested a download option to address connectivity issues: *“Students suggested a download option to address video buffering”* (FGD-S2). Concerns were also raised regarding child safety in communication features. Administrator 1 asked, *“How is safety ensured when a child communicates with someone from outside?”*

### Health-Promoting Videos Recommendations

Urdu was consistently identified as the preferred medium, valued for accessibility and emotional resonance. As Administrator 6 emphasized, *“Definitely in Urdu… no need for regional languages.”* English subtitles were recommended for higher-tier or English-medium schools.

Stakeholders agreed that videos should be short (3–7 minutes) to sustain attention. Students suggested, *“Videos should be around 5–6 minutes… explain one point clearly”* (FGD-S1)

Core content priorities included nutrition, mental health, substance abuse, physical activity, and healthy habits. Teachers highlighted dietary concerns: *“Children now consume sugary drinks and colas… harmful to health”* (FGD-T2). Administrator 1 stressed, *“Mental health is essential, especially at puberty.”* Substance abuse emerged as a major concern. Parent 3 urged, *“Add drugs alongside tobacco… include alcohol and other addictive substances.”*

In terms of format and style, participants recommended age-appropriate dramatizations. Administrator 1 noted, *“Cartoons suit younger students… for grades 9–12, use real characters or a mix.”* Administrator 2 added, *“It should be a drama or story-type video explaining diseases and prevention.”*

Stakeholders stressed the importance of clear goals, interactivity, and educational alignment to sustain engagement. Administrator 3 explained, *“You need a well-planned strategy… if it relates to their school or locality, they become more invested.”* Administrator 1 cautioned, *“If they see it as mere entertainment, they will treat it like a cartoon and forget it. Link it to their learning and SLOs [Student Learning Outcomes], and they will take it seriously.”* Teachers also emphasized age-appropriateness: *“If it’s too complex, they won’t grasp it and will lose interest”* (FGD-T1).

**Figure 4:**
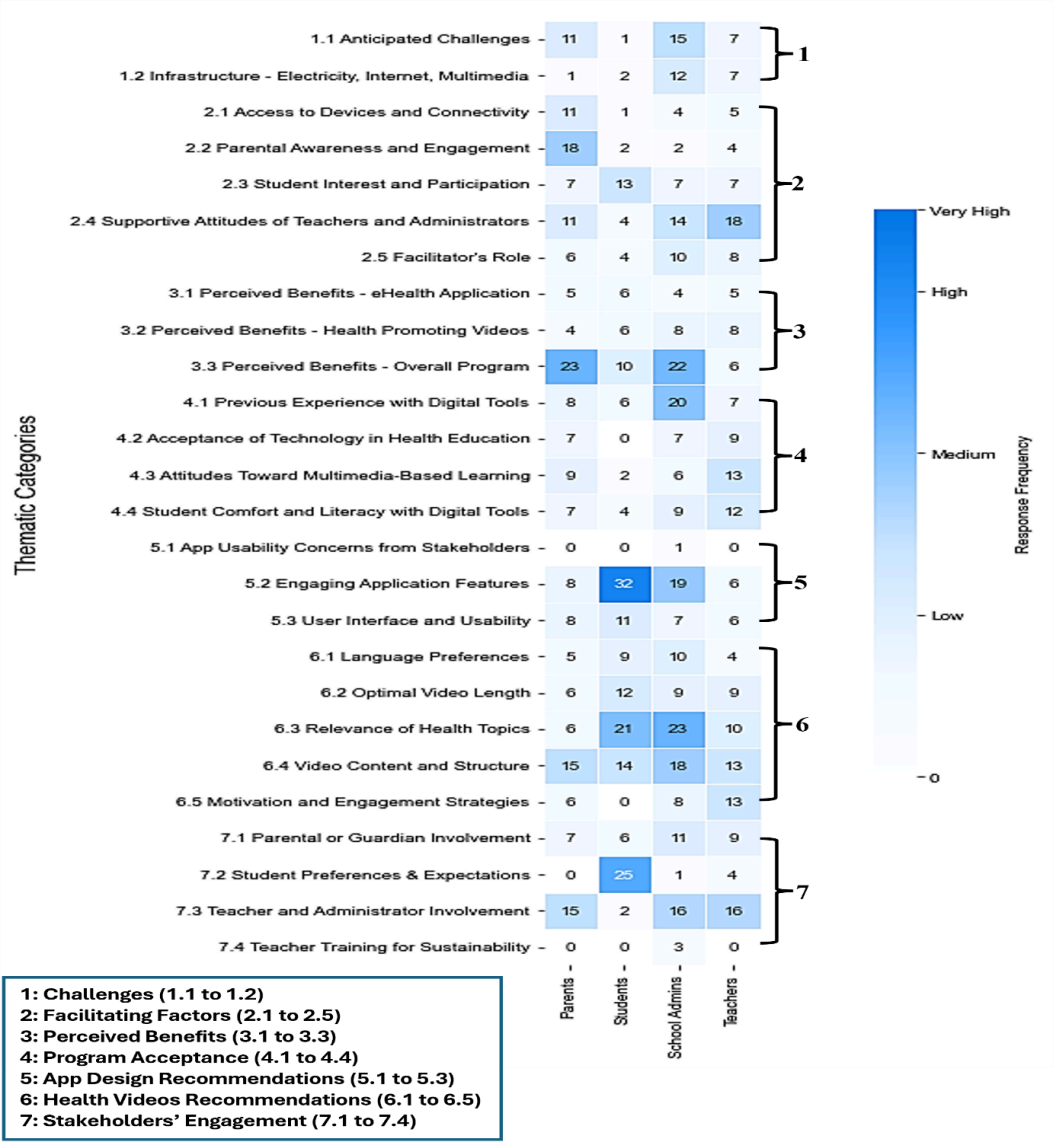
Stakeholder responses to eSHEPP themes

Figure 4 presents response frequencies from parents, students, teachers, and administrators across seven themes (challenges, facilitators, perceived benefits, program acceptance, stakeholder engagement, and recommendations for app design and health videos). Teachers emphasized support and involvement; students prioritized app design and video content; administrators and parents focused on program benefits.

## Discussion

Stakeholders noted key challenges to eSHEPP delivery, including limited infrastructure, resistance, and socio-cultural sensitivities, but also identified strong facilitators such as mobile access, parental encouragement, motivated students, and supportive teachers and administrators. They consistently emphasized stakeholder involvement and highlighted students’ readiness for digital learning, with confidence in multimedia and technology for health education.

Stakeholders recommended a simple, colorful eHealth app with Urdu/English options, intuitive navigation, offline access, privacy safeguards, and interactive features such as quizzes and rewards, alongside appealing branding. For videos, they favored short Urdu segments with English subtitles on topics such as nutrition, mental health, physical activity, and substance use, using relatable storytelling and student involvement to enhance engagement.

### Infrastructure, Access, and Digital Engagement

Stakeholders identified infrastructural barriers such as unreliable electricity, poor internet, and lack of multimedia equipment. Similar challenges are reported in other low-resource settings, including Pakistan, where few public schools have adequate digital equipment or connectivity (34–36). This aligns with national assessments of digital health in Pakistan, which highlight systemic barriers such as insufficient infrastructure, limited technical support, and fragmented implementation (37). Our findings indicate that these gaps persist at the school level, limiting the feasibility and scalability of digital health interventions.

Despite these barriers, participants reported a high level of digital familiarity among both students and teachers. Mobile phone access, particularly smartphones, was common, even in lower-income households. Students routinely accessed digital content, and teachers often used personal devices to compensate for institutional deficiencies. Such findings mirror LMIC experiences in Zimbabwe and India, where mobile-first strategies and informal device-sharing networks compensate for weak institutional infrastructure (38, 39). This contrast between weak institutional resources and strong individual digital competence underscores the potential for mobile-first eHealth programs in Pakistan, while highlighting the urgency of systemic ICT investments.

### Stakeholder Involvement and Community Acceptance

The commitment to sustain active involvement from all stakeholders, students, teachers, parents, and administrators, was a distinguishing strength of eSHEPP and the primary reason for its widespread acceptance. Students described the digital health education sessions as enjoyable and non-intrusive, teachers acknowledged their educational value, and parents endorsed the concept, demonstrating broad stakeholder support within schools. This multi-level buy-in reflects the findings of Langford et al. (2014), who emphasized that engagement from students, staff, and families is crucial for the sustainability and effectiveness of school-based health interventions (40). Comparable evidence from South Asia, such as the POD Adventures digital mental health pilot in India, shows that co-design with teachers and students enhances feasibility, acceptability, and engagement (38, 41). Our study is the first in Pakistan to demonstrate such broad, multi-level endorsement of a digital school-based health program, highlighting a strong foundation for institutionalization and scale.

Teachers and administrators played a particularly pivotal role. Their support not only facilitated student engagement but also contributed to the success of implementation efforts. This aligns with Rogers’ Diffusion of Innovations theory, which identifies early adopters as key influencers in promoting educational technologies (42). To build on this momentum, integrating digital health into pre-service and in-service teacher training could embed sustainability and ensure program continuity.

Parental support was especially pronounced in urban settings. Many parents expressed enthusiasm for app-based learning and recognized its potential to promote health literacy within households. Given the shared use of devices in many homes, students often acted as intermediaries, transferring health knowledge to family members. This finding is consistent with relational models of health education in LMICs, where adolescents often act as “knowledge brokers” who extend program reach into families and communities (43). This suggests that school-based digital interventions may also function as family-centered health promotion tools, amplifying their potential impact.

### Cultural Appropriateness and Sensitivity

Socio-cultural sensitivities, particularly around mental health, substance use, and gender norms, were a recurring theme. Evidence underscores that culturally sensitive framing and language are crucial for adolescent health interventions to gain acceptance in conservative contexts. The WHO’s 2020 youth-centered digital health framework explicitly endorses adapting content to local cultural contexts (44). In Pakistan, school-based psychological programs tailored to family and gender dynamics have shown greater feasibility than non-adapted versions (45). Similarly, South Asian review highlight how linguistically and culturally tailored digital formats improve accessibility, trust, and student participation (46). Comparable barriers have been observed in Bangladesh and India, where girls face reduced opportunities for physical activity in schools, underscoring the need for gender-sensitive strategies and community-level support (41, 47). Promoting physical activity for girls is particularly challenging due to cultural norms, limited acceptability, and restricted access to safe spaces (48).

Stakeholders preferred short Urdu-language videos with realistic scenarios and dramatizations. These were considered more relatable and emotionally resonant than abstract or overly technical presentations. Participants also emphasized avoiding fear-inducing content, preferring constructive and solution-oriented approaches. These preferences align with evidence showing that culturally tailored health communication strategies enhance both comprehension and engagement (49, 50). By foregrounding cultural sensitivity in both language and pedagogy, our study illustrates how adolescent eHealth programs can move beyond imported models to contextually rooted, locally acceptable solutions.

### Pedagogical Value and Student Empowerment

Multimedia tools, such as videos, games, and quizzes, were widely praised for their pedagogical effectiveness. Students engaged more deeply with content delivered through storytelling, gamification, and peer involvement. Evidence shows that learners generally accept and enjoy multimedia-based interventions: Donkor (2011) reported high satisfaction with video-based materials (51), while recent studies highlight how interactive formats enhance engagement and learning (52, 53). These findings align with learner-centered theories emphasizing relevance, interactivity, and active engagement (54, 55).

Teachers observed improved retention and motivation compared to traditional textbook-driven methods. Importantly, students emerged as active agents, often sharing knowledge with peers and family members. This ripple effect significantly extended the program’s reach, a pattern also reported in LMIC interventions where adolescent co-creation of content enhances ownership and sustainability (38, 56–58). This positions students not just as recipients but as co-educators, reinforcing their role in extending program impact beyond the classroom.

### Sustainability, Scale, and System Integration

While stakeholders strongly valued eSHEPP’s contribution to adolescent health education, its long-term success depends on formal integration into the education system. Sustained support from school leadership and teachers is essential for institutionalization. Stakeholders stressed the need for endorsement by education authorities and alignment with national curricula. In similar mHealth efforts in Pakistan, such as the “Hayat” system introduced in remote regions, policy integration and government funding were pivotal for scale-up beyond pilot phases (59).

Although widespread mobile phone use provides a viable delivery channel, digital inequity must be addressed for equitable access. Suggested solutions included offline access, downloadable content, and hybrid models pairing facilitators with teachers, especially in underserved areas. A recent mixed-methods analysis of digital health in Pakistan underscores systemic barriers, such as limited IT infrastructure, inconsistent connectivity, and weak regulation, that constrain scalability (37).

Training selected teachers as lead implementers with user-friendly aids was seen as key for scale. Consistent with LMIC experiences, sustainability requires strong government endorsement, integration into national priorities, and multi-stakeholder partnerships, including donor agencies (60, 61). Our findings therefore emphasize three preconditions for sustainable scale-up: investment in ICT infrastructure, incorporation into teacher training, and alignment with national education policy.

### Limitations and Future Directions

While the qualitative design provided rich contextual insights, it also introduced certain limitations. The sample, though diverse, may not fully capture the range of perspectives across Pakistan’s geographic and socio-economic landscape. Additionally, while stakeholders described observing initial signs of behavior change, longitudinal outcomes were not measured, limiting the ability to assess sustained impact.

Future research could explore the program’s application in more remote and marginalized areas and incorporate quantitative methods, such as randomized controlled trials, to assess effectiveness. Additionally, integrating app-based usage analytics and real-time feedback tools may enhance responsiveness and support iterative program improvement. More broadly, these findings offer transferable lessons for LMICs, demonstrating the value of stakeholder-driven design in bridging the “design–reality gap” and supporting contextually grounded digital health solutions.

## Conclusion

eSHEPP shows strong potential as a scalable, culturally sensitive, and pedagogically modern model for school-based health education in Pakistan. Students, teachers, parents, and administrators expressed broad support for its content and delivery, particularly its use of multimedia and interactive tools that foster engagement and align with contemporary learning approaches. Parental encouragement and administrative backing further strengthened acceptance, underscoring the importance of multi-level stakeholder involvement.

For long-term sustainability, eSHEPP must be integrated into educational structures through supportive policies, teacher training, and investment in ICT infrastructure. Addressing digital inequities, especially in underserved areas, will be essential to ensure inclusivity. With iterative refinement and institutional commitment, eSHEPP can significantly enhance adolescent health literacy and contribute to NCD prevention. These findings also provide transferable lessons for other LMICs working to embed digital health education within school systems.

## Supplementary Material

Supplementary Files

This is a list of supplementary files associated with this preprint. Click to download.

• S1File.Codebookauditandreliabilitydocumentation.docx

• S4File.COREQchecklist.docx

• S2File.Fullcodebook.docx

• S5File.Focusgroupandkeyinformantinterviewguides.docx

• S3File.TablesS4S7andFiguresS1S2.docx

## Data Availability

The deidentified interview transcripts analyzed during the current study are not publicly available due to confidentiality concerns but may be made available from the corresponding author upon reasonable request and subject to approval from the ethics committee.

## Abbreviations

AKU: Aga Khan University
COREQ: Consolidated Criteria for Reporting Qualitative Research
DEO: District Education Officer
FGD: Focus Group Discussion
ICT: Information and Communication Technology
KII: Key Informant Interview
LMICs: Low- and Middle-Income Countries
NCDs: Noncommunicable Diseases
eSHEPP: School eHealth Education Program Pakistan
SMC: School Management Committee
SLO: Student Learning Outcome
TTF: Task-Technology Fit
TAM: Technology Acceptance Model
WHO: World Health Organization
